# Supplementary material for: Discriminating neural ensemble patterns through dendritic computations in randomly connected feedforward networks
Source: eLife. 2025 Jan 24;13:RP100664. doi: 10.7554/eLife.100664 (PMC11759408; doi:10.7554/eLife.100664)
Supplement: Supplementary file 1. — (a) Molecular species used in the Ca2+-CaM model. (b) Reaction parameters of the Ca2+-CaM model. (c) Kappa values for different kinds of groups. (d) p-Values for Figure 5F. (e) p-Values for Figure 6E. (f) Parameters of the multiscale model. (g) Channel distributions used in the multiscale model. [file elife-100664-supp1.docx]

Discriminating neural ensemble patterns through dendritic computations in randomly connected feedforward networks

Bhanu Priya Somashekar

Upinder Singh Bhalla

National Centre for Biological Sciences, Tata Institute of Fundamental Research

Bangalore, Karnataka 560065, India

Correspondence: bhalla@ncbs.res.in

Lead Contact: Upinder Singh Bhalla

# Supplementary information

## Tables

| **Pools** | **Initial concentration (**μM) | **Buffered** | **Diffusion D (**μm^2^/s) |
| --- | --- | --- | --- |
| Ca | 0.08 | No | 20 |
| Ca_buffer | 0.08 | Yes | 0 |
| Ca_input | 0 | Yes | 0 |
| CaM | 2 | No | 20 |
| CaM_Ca | 0 | No | 20 |
| CaM_Ca2 | 0 | No | 20 |
| CaM_Ca3 | 0 | No | 20 |
| CaM_Ca4 | 0 | No | 20 |

1a - Molecular species used in the Ca^2+^-CaM model

| **Reactions** | **Kf** | **Kb** |
| --- | --- | --- |
| CaM + Ca <===> CaM-Ca | 7.2954 uM^-1.s^-1 | 8.4853 s^-1 |
| CaM-Ca + Ca <===> CaM-Ca2 | 7.2954 uM^-1.s^-1 | 8.4853 s^-1 |
| CaM-Ca2 + Ca <===> CaM-Ca3 | 4.2992 uM^-1.s^-1 | 10 s^-1 |
| CaM-Ca3 + Ca <===> CaM-Ca4 | 4.2992 uM^-1.s^-1 | 10 s^-1 |

1b - Reaction parameters of the Ca^2+^-CaM model

|  | 𝜿_cFMG_ | 𝜿_aFMG_ | 𝜿_cSDG_ | 𝜿_aSDG_ | 𝜿_NG_ | 𝜿_AG_ |
| --- | --- | --- | --- | --- | --- | --- |
| Hippo-chem | 2.8042 | 2.9148 | 2.6925 | 2.7582 | 2.4528 | 2.4545 |
| Hippo-CICR | 2.8042 | 2.9148 | 2.6925 | 2.7582 | 2.6857 | 2.706 |
| Hippo-elec | 3.3746 | 3.4441 | 3.6371 | 3.7011 | 3.4276 | 3.6313 |
| Cortex-chem | 2.5614 | 2.5766 | 2.63 | 2.6355 | 1.9086 | 1.5613 |
| Cortex-CICR | 2.5614 | 2.5766 | 2.63 | 2.6355 | 2.4406 | 2.2089 |
| Cortex-elec | 3.9674 | 4.0168 | 4.3914 | 4.4341 | 4.3427 | 4.6213 |

1c - Kappa values for different kinds of groups

| **Sequence Length** | **1-ectopic** | **2-ectopic** |
| --- | --- | --- |
| 3 | 0.01 | 0.002 |
| 4 | 0.002 | 0.002 |
| 5 | 0.002 | 0.002 |
| 6 | 0.037 | 0.002 |
| 7 | 0.02 | 0.002 |
| 8 | 0.002 | 0.002 |
| 9 | 0.004 | 0.002 |
| 10 | 0.02 | 0.002 |

1d - p-values for Figure 5F

| **Sequence Length** | **1-ectopic** |
| --- | --- |
| 3 | 0.996 |
| 4 | 0.996 |
| 5 | 0.002 |
| 6 | 0.002 |
| 7 | 0.002 |
| 8 | 0.002 |
| 9 | 0.002 |
| 10 | 0.002 |

1e - p-values for Figure 6E

### Integrated multiscale model parameters and equations

Chemical model: Reduced MAPK system. All volumes are initial reference volumes, and are rescaled to actual volumes defined by the detailed morphology of the simulated neuron and subsequent spatial discretization for solving PDEs.

| PSD | Vol = 0.01 fl |  |  |
| --- | --- | --- | --- |
| Reactions | Kf | Kb |  |
| Ca_input <===> Ca | 500 s^-1 | 10 s^-1 |  |
| CaM-Ca3 + Ca <===> CaM-Ca4 | 1.8 uM^-1.s^-1 | 10 s^-1 |  |
| CaM + Ca <===> CaM-Ca | 8.4846 uM^-1.s^-1 | 8.4853 s^-1 |  |
| CaM-Ca2 + Ca <===> CaM-Ca3 | 3.6001 uM^-1.s^-1 | 10 s^-1 |  |
| CaM-Ca + Ca <===> CaM-Ca2 | 8.4846 uM^-1.s^-1 | 8.4853 s^-1 |  |
|  |  |  |  |
| Pools |  |  |  |
| name | Initial Conc | buffered | D (μm^2/s) |
| Ca | 0.1 uM | No | 100 |
| Ca_input | 0.08 uM | Yes | 0 |
| CaM | 40 uM | No | 20 |
| CaM-Ca3 | 0 uM | No | 20 |
| CaM-Ca2 | 0 uM | No | 20 |
| CaM-Ca | 0 uM | No | 20 |
| CaM-Ca4 | 0 uM | No | 20 |
|  |  |  |  |
| Spine Head | Vol = 0.09 fl |  |  |
| Reactions | Kf | Kb |  |
| CaM-Ca3 + Ca <===> CaM-Ca4 | 1.8 uM^-1.s^-1 | 10 s^-1 |  |
| CaM + Ca <===> CaM-Ca | 8.4845 uM^-1.s^-1 | 8.4853 s^-1 |  |
| CaM-Ca2 + Ca <===> CaM-Ca3 | 3.6001 uM^-1.s^-1 | 10 s^-1 |  |
| CaM-Ca + Ca <===> CaM-Ca2 | 8.4845 uM^-1.s^-1 | 8.4853 s^-1 |  |
| CaM <===> CaM_xchange | 1 s^-1 | 100 s^-1 |  |
|  |  |  |  |
| Pools |  |  |  |
| name | InitialConc | buffered | D (μm^2/s) |
| Ca | 0.11111 uM | No | 100 |
| CaM | 40 uM | No | 0.5 |
| CaM-Ca3 | 0 uM | No | 1 |
| CaM-Ca2 | 0 uM | No | 1 |
| CaM-Ca | 0 uM | No | 1 |
| CaM-Ca4 | 0 uM | No | 1 |
| CaM_xchange | 0 uM | No | 20 |
|  |  |  |  |
| Dendrite | Vol = 1 fl |  |  |
| Reactions | Kf | Kb |  |
| AA <===> APC | 0.4 s^-1 | 0.01 s^-1 |  |
| 2 Ca + Raf <===> act_Raf | 12 uM^-2.s^-1 | 4 s^-1 |  |
| K_A_p <===> K_A | 0.05 s^-1 | 0 s^-1 |  |
| 2 AA + PKC <===> act_PKC | 1 uM^-2.s^-1 | 2 s^-1 |  |
| Ca_input <===> Ca | 500 s^-1 | 10 s^-1 |  |
| reg_phosphatase <===> inact_phosphatase | 0.03 s^-1 | 0 s^-1 |  |
| CaM-Ca3 + Ca <===> CaM-Ca4 | 1.8 uM^-1.s^-1 | 10 s^-1 |  |
| CaM + Ca <===> CaM-Ca | 8.4846 uM^-1.s^-1 | 8.4853 s^-1 |  |
| CaM-Ca2 + Ca <===> CaM-Ca3 | 3.6 uM^-1.s^-1 | 10 s^-1 |  |
| CaM-Ca + Ca <===> CaM-Ca2 | 8.4846 uM^-1.s^-1 | 8.4853 s^-1 |  |
| CaM <===> CaM_xchange | 10 s^-1 | 10 s^-1 |  |
|  |  |  |  |
| Enzyme-reactions | Km | kcat | ratio |
| P_MAPK ---phosphatase--> MAPK | 0.02 uM | 1 s^-1 | 4 |
| APC ---P_MAPK--> AA | 5 uM | 10 s^-1 | 4 |
| K_A ---P_MAPK--> K_A_p | 10 uM | 10 s^-1 | 4 |
| inact_phosphatase ---P_MAPK--> reg_phosphatase | 1 uM | 0.1 s^-1 | 4 |
| MAPK ---act_PKC--> P_MAPK | 5 uM | 10 s^-1 | 4 |
| MAPK ---act_Raf--> P_MAPK | 20.001 uM | 10 s^-1 | 4 |
| P_MAPK ---reg_phosphatase--> MAPK | 0.099998 uM | 2 s^-1 | 4 |
|  |  |  |  |
| Pools |  |  |  |
| name | Initial Concen | buffered | D (μm^2/s) |
| phosphatase | 0.4 uM | No | 1 |
| P_MAPK | 0 uM | No | 1 |
| MAPK | 2 uM | No | 1 |
| AA | 0 uM | No | 1 |
| act_PKC | 0 uM | No | 0 |
| PKC | 1 uM | No | 1 |
| APC | 1 uM | Yes | 0 |
| K_A | 1 uM | No | 0 |
| Raf | 1.4 uM | No | 0 |
| act_Raf | 0 uM | No | 0 |
| Ca | 0.08 uM | No | 100 |
| Ca_input | 0.08 uM | Yes | 0 |
| K_A_p | 0 uM | No | 0 |
| inact_phosphatase | 1 uM | No | 1 |
| reg_phosphatase | 0 uM | No | 1 |
| CaM | 2 uM | No | 0.5 |
| CaM-Ca3 | 0 uM | No | 1 |
| CaM-Ca2 | 0 uM | No | 1 |
| CaM-Ca | 0 uM | No | 1 |
| CaM-Ca4 | 0 uM | No | 1 |
| CaM_xchange | 0 uM | No | 20 |
|  |  |  |  |

1f - Parameters of the multiscale model

Electrical model: V in mV, referenced to resting potential. Time in ms.

Ion channel definitions, mostly from Traub, Wong, Miles, and Richardson. 1991. J. Neurophysiol 66:635-650.

gCa = gmaxCa.s^2^r ECa = 140

s-gate: $\alpha=\frac{1.6}{1+exp(-0.072\left( V-65 \right))}$ $\beta=\frac{0.02(V-51.1)}{exp\left( \frac{V-51.1}{5} \right)-1}$

r-gate for $V\leq0$: $\alpha=0.005$ $\beta=0.0$

r-gate for $V>0$: $\alpha=\frac{exp(-\frac{V}{20})}{200}$ $\beta=0.005-\alpha$

gNa = gmaxNa.m^2^h ENa = 115

m-gate: $\alpha=\frac{0.32(13.1-V)}{exp\left( \frac{13.1-V}{4} \right)-1}$ $\beta=\frac{0.28(V-40.1)}{exp\left( \frac{V-40.1}{5} \right)-1}$

h-gate: $\alpha=0.128exp(\frac{17-V}{18})$ $\beta=\frac{4}{1+exp(\frac{40-V}{5})}$

gKDR = gmaxKDR.n EKDR = -15

n-gate: $\alpha=\frac{0.016(35.1-V)}{exp\left( \frac{35.1-V}{5} \right)-1}$ $\beta=0.25exp(\frac{20-V}{40})$

gKAHP – gmaxKAHP.q EKAHP = -15

q-gate: $\alpha=min(20\times{10}^{-6}\left[ Ca \right],0.01)$ $\beta=0.001$

gKA = gmaxKA.ab EKA = -15

a_gate: $\alpha=\frac{0.02(13.1-V)}{exp\left( \frac{13.1-V}{10} \right)-1}$ $\beta=\frac{0.0175(V-40.1)}{exp\left( \frac{V-40.1}{10} \right)-1}$

b-gate: $\alpha=0.0016exp(\frac{-13-V}{18})$ $\beta=\frac{0.05}{1+exp(\frac{10.1-V}{5})}$

gKC = gmaxKC.c EKC = -15

c_gate for V <= 50: $\alpha=\frac{exp(\frac{V-10}{11}-\frac{V-6.5}{27})}{18.975}$ $\beta=2exp(\frac{6.5-V}{27})$

c_gate for V > -50: $\alpha=2exp(\frac{6.5-V}{27})$ $\beta=0$

$g_{GluR}=\frac{A.{gmax}_{GluR}}{\tau1-\tau2}(exp(\frac{-t}{\tau1)-exp(\frac{-t}{\tau2}))}$ where

A = normalization constant such that gGluR = gmaxGluR at peak, and

τ1 = 2 τ2 = 9

$g_{GABAR}=\frac{A.{gmax}_{GABAR}}{\tau1-\tau2}(exp(\frac{-t}{\tau1)-exp(\frac{-t}{\tau2}))}$ where

A = normalization constant such that gGABAR = gmaxGABAR at peak, and

τ1 = 4 τ2 = 9

$g_{NMDAR}=\frac{{gmax}_{NMDAR}}{\tau}exp(\frac{-t}{\tau)\frac{K_{Mg}}{K_{Mg}+\left[ Mg \right]}}$ where

$K_{Mg}=exp\frac{\left( (V-Erest)\gamma\right)}{\eta}$ τ = 20

γ = 0.28 η = 62

$I_{{NMDAR}_{Ca}}=g_{NMDAR}.{Ca}_{\frac{}{.}ln(\frac{\left[ {Ca}_{out} \right]}{\left[ {Ca} \right]).V.\frac{\left[ {Ca} \right]-\varphi\left[ {Ca}_{out} \right]}{(1-\varphi)(\left[ {Ca} \right]-\left[ {Ca}_{out} \right])}}}$ where

$\varphi=exp(\frac{-VFz}{RT})$ F = 96485 sA/mol

z = 2 R = 8.314 J/(K.mol)

T = 300 Kelvin Ca_frac_= fraction of current carried at 0 mV by Ca = 0.02

[Ca_out] = 1.5mM [Ca_in] = 0.08 μM

Calcium pools:

d[Ca]/dt = φ(ICa + CaNMDA) –[Ca]/13.33

Passive properties

RM = 1.0 Ω.m^2^ RA = 1.0 Ω.m CM = 0.01 F/m^2^

Erest = -70 mV.

Channel distributions

| Channel | Zone | Distribution, i.e., Gmax. (S/m^2^) |
| --- | --- | --- |
| Ca | Apical | 8 |
| Ca | Soma | 40 |
| Na | Basal | 60 |
| Na | Apical | 40+40exp(-p/200) |
| Na | Soma | 600 |
| K_DR | Basal | (p<400)*200 |
| K_DR | Apical | 60+40exp(p<125) |
| K_DR | Soma | 360 |
| K_AHP | All | 8 |
| K_C | Apical | 50+150exp(-p/200) |
| K_C | Soma | 100 |
| K_A | Apical | 50(1+2/(dia+0.1)) |
| K_A | Soma | 50 |
| GABA | All | 10+30(p < 125) |
| GluR | Spine Heads | 4000 |
| NMDAR | Spine Heads | 800 |

1g - Channel distributions used in the multiscale model

Where p = path length in microns measured along dendrite, from soma to specified point on dendritic tree, and

dia = diameter of dendrite at specified point on the dendritic tree.
